# Prediction of Insulin Resistance and Impaired Fasting Glucose Based on Sex Hormone-Binding Globulin (SHBG) Levels in Polycystic Ovary Syndrome

**DOI:** 10.1155/2022/6498768

**Published:** 2022-01-31

**Authors:** Aleksandra Biernacka-Bartnik, Piotr Kocełak, Aleksander Jerzy Owczarek, Piotr Choręza, Monika Puzianowska-Kuźnicka, Leszek Markuszewski, Paweł Madej, Jerzy Chudek, Magdalena Olszanecka-Glinianowicz

**Affiliations:** ^1^Department of Gynecological Endocrinology, Faculty of Medical Sciences in Katowice, Medical University of Silesia in Katowice, Katowice, Poland; ^2^Pathophysiology Unit, Department of Pathophysiology, Faculty of Medical Sciences in Katowice, Medical University of Silesia in Katowice, Katowice, Poland; ^3^Health Promotion and Obesity Management Unit, Department of Pathophysiology, Faculty of Medical Sciences in Katowice, Medical University of Silesia in Katowice, Katowice, Poland; ^4^Department of Statistics, Department of Instrumental Analysis, Faculty of Pharmaceutical Sciences in Sosnowiec, Medical University of Silesia, Katowice, Poland; ^5^Department of Human Epigenetics, Mossakowski Medical Research Institute, Polish Academy of Sciences, Warsaw, Poland; ^6^Department of Geriatrics and Gerontology, Medical Centre of Postgraduate Education, Warsaw, Poland; ^7^Center Heart and Vascular Diseases Internal and Metabolic Diseases Mazovian Specialist Hospital in Radom, University of Humanities and Technology in Radom, Radom, Poland; ^8^Department of Internal Medicine and Oncological Chemotherapy, Faculty of Medical Sciences in Katowice, Medical University of Silesia in Katowice, Katowice, Poland

## Abstract

**Objective:**

Decreased synthesis of sex hormone-binding globulin (SHBG) related to hyperinsulinemia is one of the disturbances characteristic of polycystic ovary syndrome (PCOS). Hyperinsulinemia is a compensatory mechanism for liver insulin resistance (IR); thus, SHBG may be considered as a surrogate marker of liver IR. Therefore, this study aimed to assess the prediction of IR and impaired fasting glucose (IFG) based on SHBG levels in women with PCOS.

**Methods:**

This analysis included data retrieved from medical records of 854 patients with PCOS hospitalized in the Gynecological Endocrinology Clinic from 2012 to 2019. Data including anthropometric parameters, fasting plasma glucose, insulin, and SHBG levels were analyzed. BMI and HOMA-IR were calculated with standard formulas.

**Results:**

IFG and IR assessed based on HOMA-IR values > 2.0 were found in 19.5% and 47.8% of the study group, respectively. Empirical optimal cutoff values for SHBG levels were ≤41.5 nmol/L typical for IR (AUC 0.711, sensitivity 61.1%, specificity 71.6%, positive predictive value (PPV) 70.7%, and negative predictive value (NPV) 62.1%). The probability of insulin resistance occurrence for SHBG concentration 26.1 nmol/L (the lower normal range) was 61.6% (95% CI: 57.4%–65.8%). The SHBG concentration of 36.4 nmol/L and 8.1 nmol/L was related to a 10% and 20% probability of IFG, respectively.

**Conclusion:**

In conclusion, this is the first study estimating the probability of liver IR and IFG occurrence based on SHBG levels in women with PCOS. Despite the low sensitivity, SHBG level below 42 nmol/L should cause closer monitoring for the fatty liver and prediabetes.

## 1. Introduction

According to some recent studies, obesity, especially visceral obesity, is an important factor contributing to hormonal and metabolic disturbances in polycystic ovary syndrome (PCOS) [1,2]. Increased volume of adipocytes causes an influx of macrophages into visceral adipose tissue and stimulates the production of proinflammatory cytokines. Emerging low-grade chronic inflammation induces hormonal disturbances of adipose tissue and insulin resistance (IR) initially locally and then in the liver and muscles [3,4]. The increased insulin level, a compensatory mechanism for liver and insulin resistance, as well as the decreased circulating SHBG level are typical for PCOS. Notably, hyperinsulinemia is one of the factors inhibiting SHBG synthesis in the liver. Therefore, SHBG may be considered as a surrogate marker of hepatic insulin resistance [5–7].

The occurrence of impaired glucose tolerance (IGT) in women with PCOS is estimated at 31–35% while in the general population of women of reproductive age at 1.6%. Similarly, type 2 diabetes occurs in 7.5–10% and 2.2% of women, respectively [8–10]. It has been shown that the 2-year risk of developing prediabetes and type 2 diabetes in PCOS women with normal glucose tolerance is 2.5-fold higher than in women without this syndrome [11].

A retrospective analysis of a large primary-care cohort of 63,120 women with PCOS and 121,064 women without this syndrome revealed a more frequent occurrence of nonalcoholic fatty liver disease (NAFLD) among PCOS women. Moreover, SHBG < 30 nmol/L and testosterone > 3 nmol/L were associated with a higher incidence of NAFLD [12]. The association between lower SHBG levels and the occurrence of NAFLD was confirmed in a meta-analysis of 16 studies including 13,721 men and 5,840 women. It should be noted that this relationship was stronger in women than in men [13]. Enhanced gluconeogenesis resulting in impaired fasting glucose (IFG) is typical for NAFLD. In a cohort of 2,654 men, lower SHBG levels were associated with prediabetes status [14]. Moreover, in the 5-year follow-up of 2,077 women, SHBG concentrations were inversely associated with the risk of development of type 2 diabetes [15]. Furthermore, another 5-year follow-up study (*Environment, Inflammation, and Metabolic Diseases Study*) showed that low SHBG levels were associated with 4-fold higher risk of developing type 2 diabetes both in men and women. The risk was decreasing with the increase in SHBG tertile after multifactorial adjustment in women [16]. The association between low SHBG levels and increased risk of development of type 2 diabetes was supported by the Rotterdam study including 3,177 postmenopausal women, with a median observation of 11.1 years [17]. Also, a 6-year follow-up study that included 1,377 subjects showed that SHBG level is a marker of insulin resistance development [18]. O'Reilly et al. [19] have shown that the increased risk of developing type 2 diabetes occurred in men with SHBG <40 nmol/L and in women with SHBG < 50 nmol/L. Some other studies found low SHBG levels being the risk factor for gestational diabetes [20–23]. Hedderson et al. [21] observed that SHBG levels below the median (<64.5 nmol/L) and BMI > 25 kg/m^2^ were associated with a 5-fold increase in the risk of development of gestational diabetes. On the other hand, in a study of 180 pregnant women with PCOS, an increase in SHBG concentration by 1 nmol/L reduced the risk of developing gestational diabetes by 7% [22]. Finally, the results of a meta-analysis of 26 studies showed that SHBG testing in early pregnancy may be a marker of the risk of developing gestational diabetes [23].

Based on the abovedescribed studies, we hypothesized that SHBG levels may serve as a surrogate marker of hepatic insulin resistance in PCOS women. Therefore, this study aimed to assess the value of prediction of insulin resistance and impaired fasting glucose based on SHBG level in women with PCOS.

## 2. Materials and Methods

This retrospective study includes data from the medical records of 854 Caucasian female patients of the Gynecological Endocrinology Clinic for the first time diagnosed with PCOS based on the Rotterdam criteria during index hospitalization that took place between 2012 and 2019.

The inclusion criteria comprised age 18–30 years and diagnosis of PCOS. The exclusion criteria were diagnosis of type 2 diabetes and other endocrinological disturbances, any currently ongoing pharmacological therapy and past bariatric treatment of obesity, and the lack of completeness of necessary datasets in the medical records.

The analyzed dataset included age, body mass, height, and routine measurements of fasting glucose, insulin, and SHBG levels, all performed in a single hospital laboratory using the same set of methods for all study subjects. Glucose concentration was measured using the colorimetric method (Roche Diagnostic GmbH, Mannheim, Germany). Insulin and SHBG levels were determined using the ECLIA method (Roche, reagents for Cobas E411). BMI and HOMA-IR values were calculated with standard formulas.

The retrospective analysis did not fulfill the criteria of a medical experiment and, therefore, did not require the consent of a bioethical committee and the patient. Personal data were not proceeded in the analysis.

### 2.1. Data Analysis

The study group was divided according to an HOMA-IR cutoff value of 2.0 [24] into the subgroups with (*N* = 408; 47.8%) and without insulin resistance (*N* = 446; 52.2%).

### 2.2. Statistical Analysis

Statistical analysis was performed using STATISTICA 13.0 PL (TIBCO Software Inc., Palo Alto, CA, U.S.), StataSE 13.0 (StataCorp LP, TX, U.S.), and R software (R Core Team (2013). R: a language and environment for statistical computing, R Foundation for Statistical Computing, Vienna, Austria, URL http://www.r-project.org/. Statistical significance was set at a *p* value below 0.05. All tests were two tailed. Imputations were not performed for missing data. Nominal and ordinal data were expressed as percentages. Interval data were expressed as mean ± standard deviation in case of normal data distribution or as median (lower-upper quartiles) in data with nonnormal or skewed distribution. The distribution of variables was evaluated by the Shapiro–Wilk W test and the quantile-quantile (Q-Q) plot. To compare two groups with HOMA ≥ 2.0 and HOMA < 2.0, the Student's *t*-test for independent data or the *U* Mann–Whitney test was used, according to data distribution, and homogeneity of variances was assessed by the Fisher–Snedecor F test. To find a cutoff point discriminating the insulin resistance and impaired fasting glucose based on the SHBG level, parametric and nonparametric ROC curves were calculated with the area under curve (AUC) and corresponding sensitivity, specificity, and positive and negative predictive values. The fractional polynomial curve with a 95% confidence interval was calculated to show the associations between SHBG levels and insulin levels. The logistic regression was used to assess the probability of IR and IFG based on the SHBG levels.

## 3. Results

Insulin resistance assessed based on HOMA-IR values 2.0 and above was found in 47.8% (*N* = 408) of the study group and IFG in 9.4% (*N* = 80). Compared to patients without IR, the IR group was characterized by significantly higher BMI values and more frequent occurrence of obesity and significantly higher fasting glucose and insulin concentrations, as well as more common IFG, lower median SHBG levels (30.8 vs. 49.1 nmol/L; *p* < 0.001), and a more frequent occurrence of SHBG concentration below laboratory's lower limit of the normal range for women aged 18–50 years (<26.1 nmol/L). The detailed study groups characteristics are listed in Table 1.

**Figure 1:**
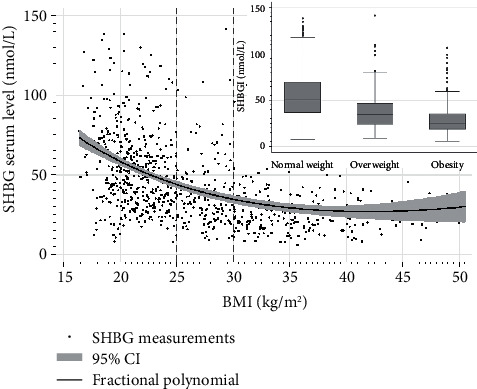
SHBG levels depending on nutritional status.

As expected, the median SHBG level was the lowest in the subgroup with obesity and the highest in the normal weight subgroup (Table 2 and Figure 1). On the contrary, glucose and insulin levels were the highest in the subgroup with obesity and the lowest in the normal weight subgroup. It addition, the percentage of women with insulin resistance and with SHBG level below the lower limit of the reference range (<26.1 nmol/L) was the highest in the subgroup with obesity and the lowest in the normal weight subgroup (Table 2).

**Figure 2:**
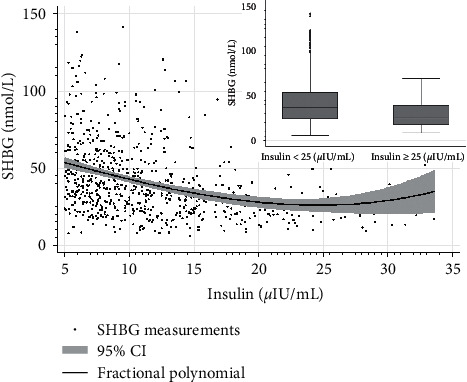
SHBG levels depending on insulin levels.

The median SHBG levels were significantly higher in patients with normal insulin levels than in these with levels above laboratory's reference range (>25 *μ*IU/mL) (36.9 nmol/L (24.4–53.8) vs. 25.3 nmol/L (17.6–39.5); *p* < 0.01) (Figure 2). Empirical optimal cutoff values for SHBG levels, based on the ROC analysis, that characterize individuals with IR were ≤41.5 nmol/L (AUC 0.71, sensitivity 61.1%, specificity 71.6%, positive predictive value (PPV) 70.7%, and negative predictive value (NPV) 62.1%), Table 3.

**Figure 3:**
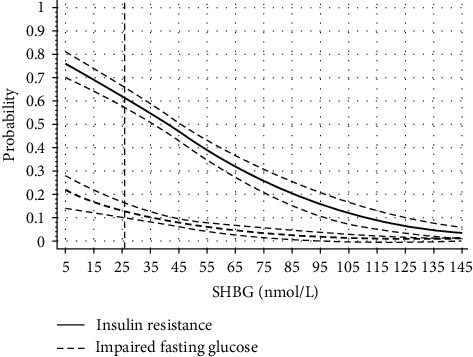
The probability of insulin resistance and impaired fasting glucose occurrence depending on SHBG levels.

We observed 10% probability of insulin resistance occurrence at an SHBG concentration of 111.1 nmol/L, 20% at 85.3 nmol/L, 25% at 76.1 nmol/L, 33.3% at 63.2 nmol/L, and 50% at 41.1 nmol/L, while the probability of IR for an SHBG concentration of 26.1 nmol/L was 61.6% (95% CI: 57.4–65.8). Also, there was a 10% probability of IFG occurrence at an SHBG concentration 36.4 nmol/L and 20% at 8.1 nmol/L. The probability of IR at SHBG concentration 36.4 nmol/L was 53.7% (95% CI: 50.0–57.4) and at 8.1 nmol/L 73.8% (95% CI: 68.7–79.1) (Figure 3).

## 4. Discussion

To the best of our knowledge, this is the first study that estimates the probability of insulin resistance and impaired fasting glucose occurrence in women with PCOS according to cutoff values for SHBG levels.

Low circulating SHBG levels are associated with the development of type 2 and gestational diabetes [14–19, 25, 26]. The results of a 15-year follow-up study suggested that a decreased synthesis of SHBG in the liver is an early response to the increased insulin levels corresponding to IR in visceral fat [27]. The mechanism of SHBG synthesis inhibition was linked with leptin [28, 29].

The HOMA-IR is widely used for the assessment of insulin resistance better reflecting hepatic than muscle insulin resistance [30]. In our study, the cutoff HOMA-IR value for diagnosis of insulin resistance was set at 2.0. As expected, SHBG levels were significantly lower in the insulin-resistant patients, and in this group, SHBG levels below the laboratory's reference range for women aged 18–50 years (<26.1 nmol/L) and IFG were more frequently present than in patients without IR.

We observed a 50% probability of IR occurrence at an SHBG concentration of 41.1 nmol/L. It is consistent with the results of the study enrolling 42,034 women in whom the risk of development of type 2 diabetes increased by 299% when SHBG levels were in the range from 40 to 49.99 nmol/L [19] and the study showing that the risk of gestational diabetes development increased five-fold with median SHBG levels below 64.5 nmol/L [21]. However, an SHBG concentration of 111.1 nmol/L was already associated with a 10% probability of IR. In our study, based on the ROC analysis, the empirical optimal SHBG cutoff point for insulin resistance was 41.5 nmol/L. This SHBG cutoff point is characterized by lower sensitivity yet higher specificity, with higher positive than negative predictive value. In addition, AUC illustrating the diagnostic power of the test of 0.711 was lower than accepted for most diagnostic tests in the range of 0.80–0.95. Therefore, one should be cautious about the assumption that SHBG concentrations may replace other markers for assessing insulin resistance. However, the results of our study indicate that SHBG concentrations < 42 nmol/L, higher than the values adopted as the lower limit of normal laboratory range < 26.1 nmol/L, should lead to diagnostics for impaired fasting glucose (if it has not already been carried out), as a group of young PCOS women are already at the increased risk of their occurrence. On the other hand, an SHBG concentration of 36.4 nmol/L was associated with 53.7% probability of IR and a low, 10% probability of IFG. However, in our cohort, fasting glucose measurement was performed only once and the already existing IFG may have been overlooked, causing such a large discrepancy. This hypothesis is supported by results obtained by Jaygobal et al. [31] who assessed the biological variability of fasting glucose, insulin, and SHBG in 10 samples obtained every 4 days from the same individuals. This study showed that glucose and insulin levels present significant variability which, in addition, was much higher than the variability of SHBG levels. In addition, the results of a meta-analysis of 28 studies conducted in women with and without PCOS (*n* = 741 and *n* = 1224, respectively) showed that a 10 unit higher BMI reduces insulin sensitivity as assessed by euglycemic-hyperinsulinemic clamp by 28% and 15%, respectively, and low SHBG levels are independently associated with a decrease in insulin sensitivity [32]. The authors of this meta-analysis indicate the need to search cutoff points for SHBG levels to diagnose IR, which was the subject of our study.

IFG is a clinical symptom of liver IR. However, it is not known how early it occurs; therefore, SHBG levels may be considered as an early marker of liver IR preceding IFG. It should be also noted that the SHBG cutoff point estimated empirically in our study is an intermediate value between the cutoff point for SHBG levels associated with the risk of fatty liver in PCOS women (below 30 nmol/L) [12] and associated with the increased risk of type 2 diabetes development (below 50 mmol/L) [19]. Thus, despite the low AUC and sensitivity, the cutoff point established by us may have a clinical significance in the screening towards IR in PCOS women. Our study suggests that the given SHBG levels should initiate diagnostics and treatment of fatty liver to prevent the development of prediabetes. As mentioned above, fatty liver and liver IR result in increased gluconeogenesis and impaired fasting glucose levels. However, it should be noted that low SHBG is not an established diagnostic marker of NAFLD. Despite it, finding low SHBG level should prompt further clinical review and recommendation of standard diagnostic tests for NAFLD.

Therefore, overweight or obese women with low SHBG should be counselled with lifestyle intervention, behavioral therapy, and, in individual cases, psychotherapy and pharmacotherapy. It was indeed shown that maintaining a reduced body mass for 18 months resulted in the increase of SHBG [33].

Results of our and other studies suggest the need for a shift of the lower limit of the laboratory reference range for SHBG towards higher value. It should be taken into account that the normal ranges and units of SHBG concentration are evaluated by different methods (ECLIA, RIA, and ELISA) and may differ in the determination of the new cutoff points.

The main limitation of the present study is the lack of euglycemic-hyperinsulinemic clamp and assessment of fatty liver, as well as recognized diagnostic tests for NAFLD because NAFLD is a cause of liver insulin resistance development and decreased SHBG synthesis. It should also be noted that decreased SHBG level is an obesity-related disturbance linked to the development of liver IR, and therefore, nutritional status is one of factors indirectly affecting SHBG synthesis. Moreover, we replaced the generally accepted HOMA-IR cutoff point 2.5 for the assessment of IR in women with PCOS in a Polish population by the cutoff 2.0 calculated in our previous analysis performed in this population. On the other hand, a strength of our study is the large size of the study group and the inclusion of a homogenous group of young Caucasian women diagnosed with PCOS, with different nutritional statuses.

## 5. Conclusions

In conclusion, this is the first study estimating the probability of liver insulin resistance and impaired fasting glucose occurrence based on SHBG levels in women with PCOS. Despite the low sensitivity, the SHBG level below 42 nmol/L should result in closer monitoring for the fatty liver and prediabetes.

## Figures and Tables

**Table 1:** The study group characteristics.

	HOMA < 2.0, *N* = 446 (52.2%)	HOMA > 2.0, *N* = 408 (47.8%)	*p*
Age (years)	26 ± 5	26 ± 6	0.91
BMI (kg/m^2^)	23.0 ± 4.4	30.5 ± 7.9	<0.001
Overweight (*N*; %)	68; 15.2	81; 19.8	0.08
Obesity (*N*; %)	35; 7.8	211; 51.7	<0.001
Glucose (mg/dL)	86.0 (81.0–90.0)	91.0 (86.0–97.0)	<0.001
Impaired fasting glucose (*N*; %)	4; 0.9	76; 18.6	<0.001
Insulin (*μ*IU/mL)	6.1 (4.7–7.6)	13.3 (10.9–18.2)	<0.001
SHBG (nmol/L)	49.1 (34.1–68.2)	30.8 (20.7–44.5)	<0.001
SHBG < 26.1 (nmol/L) (*N*; %)	55; 12.3	160; 39.2	<0.001
OR impaired fasting glucose	Ref.	25.3 (9.1–69.9)	<0.001
OR SHBG < 26.1 (nmol/L)	Ref.	4.56 (3.23–6.45)	<0.001

Mean ± standard deviation or median (lower quartile–upper quartile) or 95% confidence interval for OR.

**Table 2:** The study subgroups stratified according the BMI categories.

	Normal weight, *N* = 459 (53.8%)	Overweight, *N* = 149 (17.4%)	Obesity, *N* = 246 (20.8%)	*p*
BMI (kg/m^2^)	21.1 (19.7–22.8)	27.5 (26.3–28.7)	34.8 (32.0–39.8)	—
Age (years)	25 (21–29)	25 (22–28)	26 (22–31)	0.28
Glucose (mg/dL)	87 (82–91)	88 (83–93)	91 (86–97)	<0.001
IFG (*N*; %)	20; 4.4	12; 8.1	48; 19.5	<0.001
Insulin (uIU/mL)	6.7 (4.9–9.2)	9.5 (7.4–12.2)	14.9 (11.2–20.8)	<0.001
HOMA-IR	1.4 (1.0–2.0)	2.1 (1.5–2.6)	3.5 (2.4–4.8)	<0.001
HOMA-IR ≥ 2 (*N*; %)	116; 25.3	81; 54.4	211; 85.8	<0.001
SHBG (nmol/L)	51.8 (36.6–70.7)	34.2 (24.0–46.8)	25.4 (18.7–35.4)	<0.001
SHBG < 26.1 (*N*; %)	43; 9.4	45; 30.4	127; 51.6	<0.001

Median (lower quartile–upper quartile).

**Table 3:** Sensitivity, specificity, positive predictive value, negative predictive value, and accuracy of HOMA-IR > 2.0 corresponding to low circulating SHBG levels in PCOS women.

Parameter	Value	95% CI
Sensitivity	61.1%	56.5–65.6%
Specificity	71.6%	66.9–75.8%
Positive predictive value	70.7%	65.9–75.1%
Negative predictive value	62.1%	57.6–66.5%
AUC	0.711	0.677–0.745

AUC: area under curve; CI: confidence interval.

## Data Availability

Data are available from the corresponding author on request.
